# Supplementary material for: Legionella metaeffector MavL reverses ubiquitin ADP-ribosylation via a conserved arginine-specific macrodomain
Source: Nat Commun. 2024 Mar 19;15:2452. doi: 10.1038/s41467-024-46649-2 (PMC10951314; doi:10.1038/s41467-024-46649-2)
Supplement: Supplementary file 1 — Supplementary Information [file 41467_2024_46649_MOESM1_ESM.pdf]

Legionella metaeffector MavL reverses ubiquitin ADP-ribosylation via a conserved arginine-specific macrodomain

Zhengrui Zhang[1], Jiaqi Fu[2], Johannes Gregor Matthias Rack[3,4], Chuang Li[2], Jim Voorneveld[5], Dmitri V. Filippov[5], Ivan Ahel[3], Zhao-Qing Luo[2], and Chittaranjan Das[1,*]

Chittaranjan Das
Email: cdas@purdue.edu

Supplementary information

Supplementary Figures 1 to 6
Supplementary Tables 1 to 3
Supplementary References

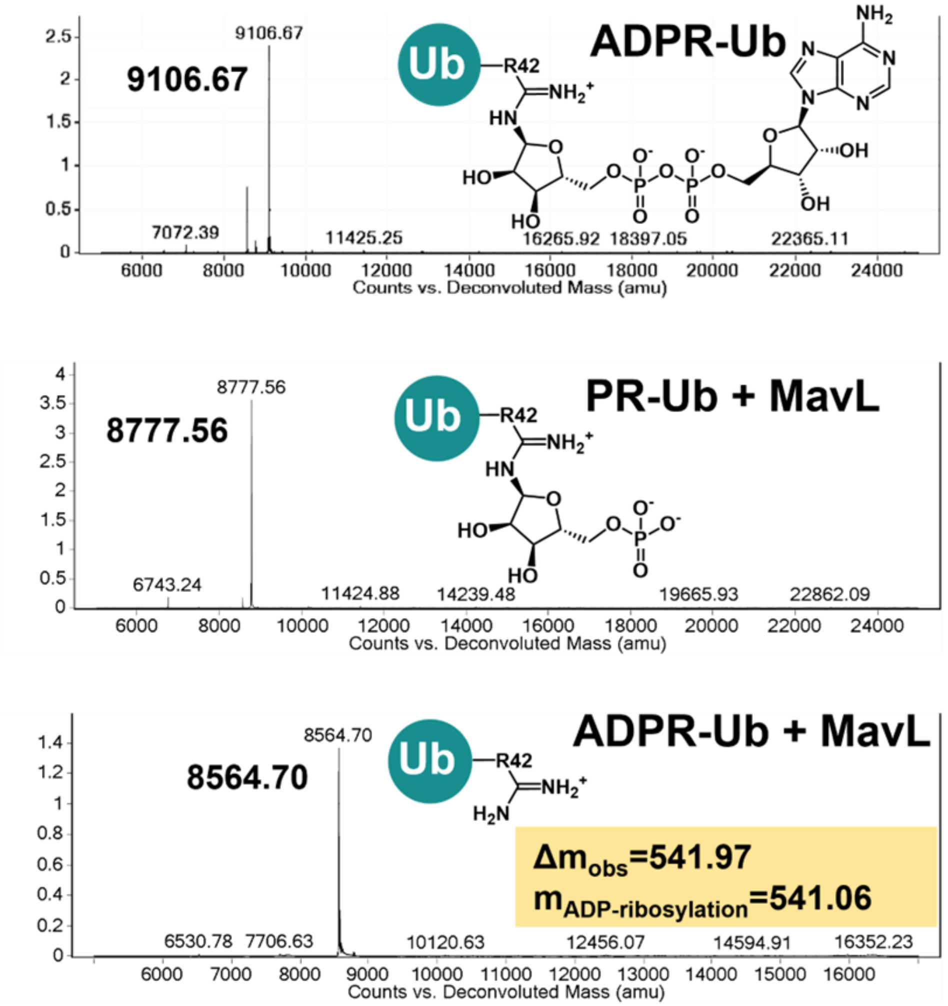

Supplementary Figure 1. Electrospray ionization mass spectrometry (ESI-MS) determination of the total mass of ADPR-Ub, PR-Ub + MavL, and ADPR-Ub + MavL. Introduction of MavL to ADPR-Ub results in a product mass corresponding to native Ub. The 541.97 Da mass loss corresponds to mass change of ADP-ribosylation.

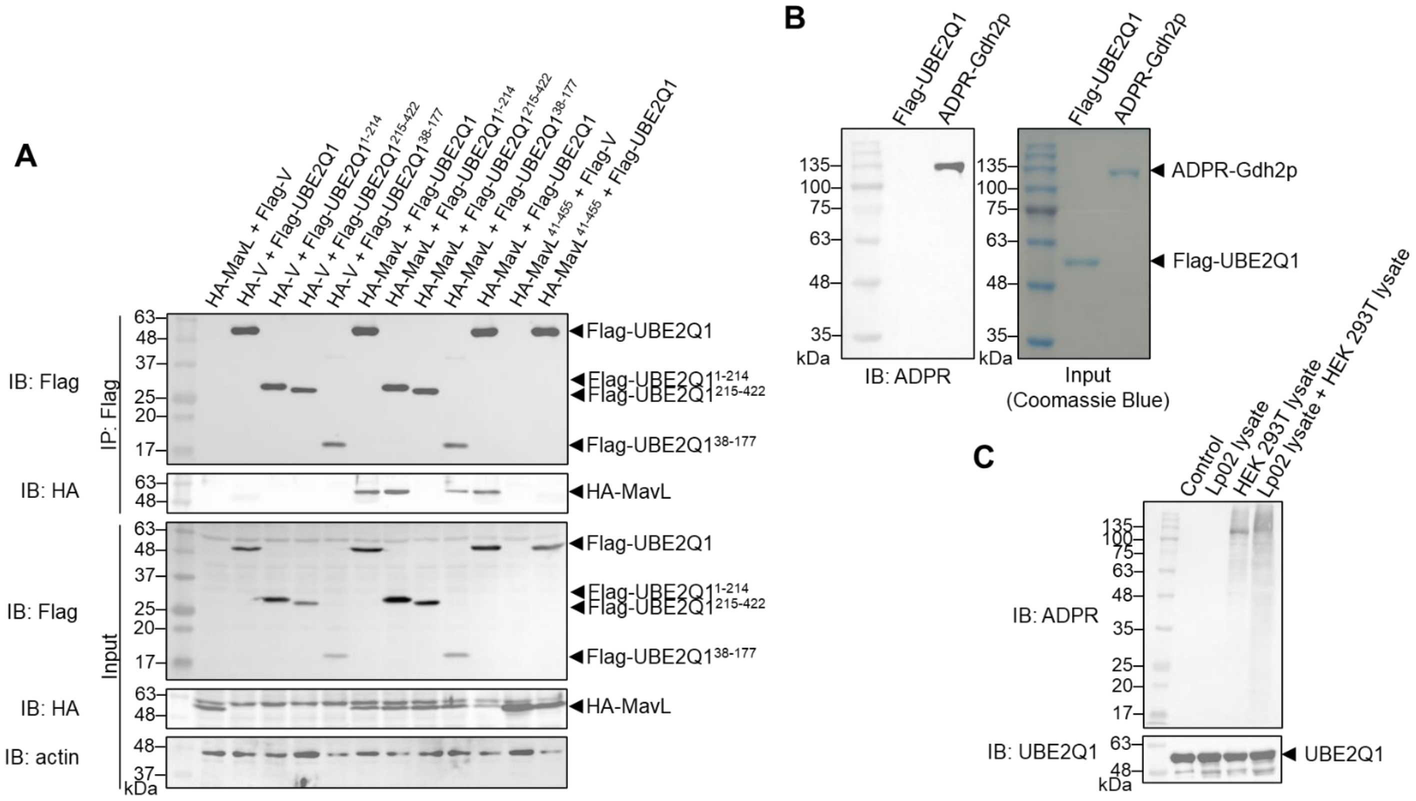

Supplementary Figure 2. Interaction between MavL and UBE2Q1. (A) Co-immunoprecipitation showing interactions between MavL and UBE2Q1. HEK293T cells were transfected to express Flag-UBE2Q1 (or truncated constructs) and HA-MavL (or N-terminus truncated construct). The cell lysate was IP-ed against Flag-tag. Expression of UBE2Q1 and MavL is included with empty vector controls (indicated as "V"), with actin as a loading control. Experiments were performed three times independently with similar results. (B) Detection of ADP-ribosylation of UBE2Q1. Flag-UBE2Q1 was ectopically expressed in HEK293T cells and enriched via immunoprecipitation. The ADP-ribosylation state was tested via immunoblotting. ADPR-Gdh2p was run as a positive control. Experiments were performed three times independently with similar results. (C) UBE2Q1 cannot be ADP-ribosylated by *L. pneumophila* lysate or HEK293T cell lysate. Recombinantly purified UBE2Q1 was co-incubated with *L. pneumophila* lysate and/or HEK293T cell lysate with NAD^+. These reactions were probed via immunoblotting against ADP-ribosylation. Loading of UBE2Q1 was shown. Experiments were performed three times independently with similar results.

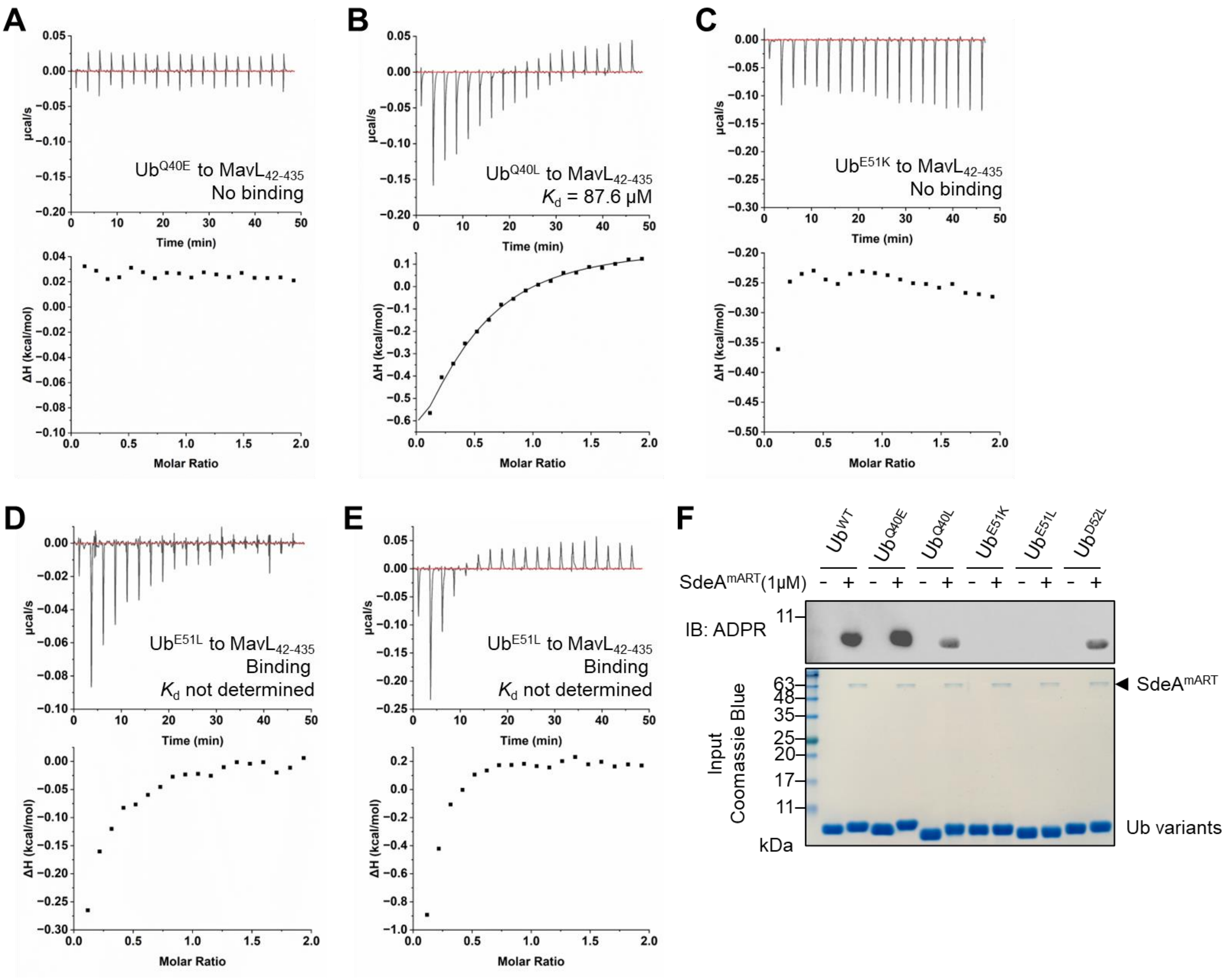

Supplementary Figure 3. MavL binding and ADP-ribosylation of Ub mutants. (A-E) Isothermal titration calorimetry profile of (A) Ub^{Q40E} to $MavL_{42-435}$, (B) Ub^{Q40L} to $MavL_{42-435}$, (C) Ub^{E51K} to $MavL_{42-435}$, (D) Ub^{E51L} to $MavL_{42-435}$, and (E) Ub^{D52L} to $MavL_{42-435}$. Raw data were integrated and fitted using a one binding site model to determine the K_d. (F) ADP-ribosylation of Ub mutants by $SdeA^{mART}$. ADPR-Ub was detected by immunoblotting against ADPR. Loading of the reactions was visualized by Coomassie Blue staining. Experiments were performed three times independently with similar results.

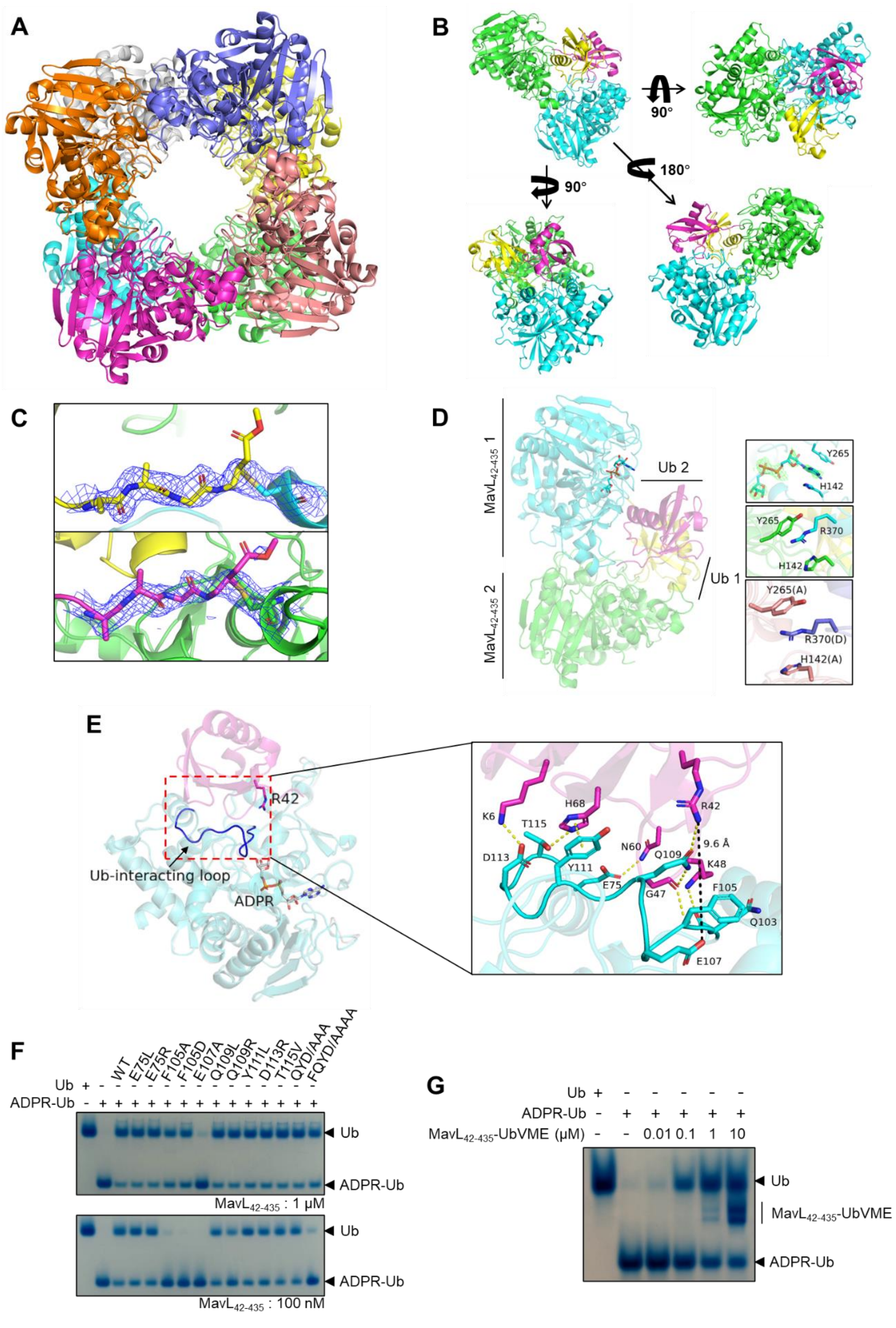
A
B
90°
180°
90°
C
D
$MavL_{42-435}$ 1
$MavL_{42-435}$ 2
Ub 2
Ub 1
Y265
H142
Y265
R370
H142
Y265(A)
R370(D)
H142(A)
E
R42
Ub-interacting loop
ADPR
K6
T115
H68
D113
Y111
E75
N60
Q109
G47
R42
9.6 Å
K48
F105
Q103
E107
F
Ub
ADPR-Ub
WT
E75L
E75R
F105A
F105D
E107A
Q109L
Q109R
Y111L
D113R
T115V
QYD/AAA
FQYD/AAAA
Ub
ADPR-Ub
$MavL_{42-435}$: 1 μM
Ub
ADPR-Ub
$MavL_{42-435}$: 100 nM
G
Ub
ADPR-Ub
$MavL_{42-435}$-UbVME (μM)
0.01
0.1
1
10
Ub
$MavL_{42-435}$-UbVME
ADPR-Ub

Supplementary Figure 4. Structrual and biochemical analysis of MavL-UbVME. (A) Overall structure of apo MavL. Eight MavL molecules were crystallized in one asymmetric unit. (B) Overall structure of MavL-UbVME complex. (C) Electron density ($2F_o$-F_c map, contour = 1σ) of covalent linkages between Ub C-terminus and MavL C226. (D) Structure of ADPR-soaked MavL-UbVME. Top right figure: Electron density of ADPR in the F_o-F_c map (contour = 3σ). Middle right figure: H142-R370-Y265 crystal packing effect observed in the second MavL subunit. R370 from one MavL (green) points between H142 and Y265 from the other MavL (cyan), occupying the space for adenosine. Bottom right figure: H142-R370-Y265 crystal packing in apo MavL, exemplified by chain A (orange) and chain D (black). (E) Left: Superposition of MavL-UbVME structure (MavL1 and Ub2) to ADPR-bound $MavL_{42\text{-}435}^{R370A}$. Ub R42, Ub-interacting loop, and ADPR were marked to show relative position of these elements. Right: MavL-Ub interface in MavL-UbVME structure showing direct interactions of Ub R42 with MavL. The MavL loop spanning Q103 to T115 makes multiple interactions with the covalently captured ubiquitin, including R42. The distance between Ub R42 and the MavL E107 is marked as a black dashed line. (F) Native-PAGE showing (ADP-ribosyl)hydrolase activity of MavL mutants based on MavL-Ub interface observed in MavL-UbVME structure. Experiments were performed three times independently with similar results. (G) (ADP-ribosyl)hydrolysis of ADPR-Ub by MavL-UbVME suggests a comparable activity to MavL. Experiments were performed three times independently with similar results.

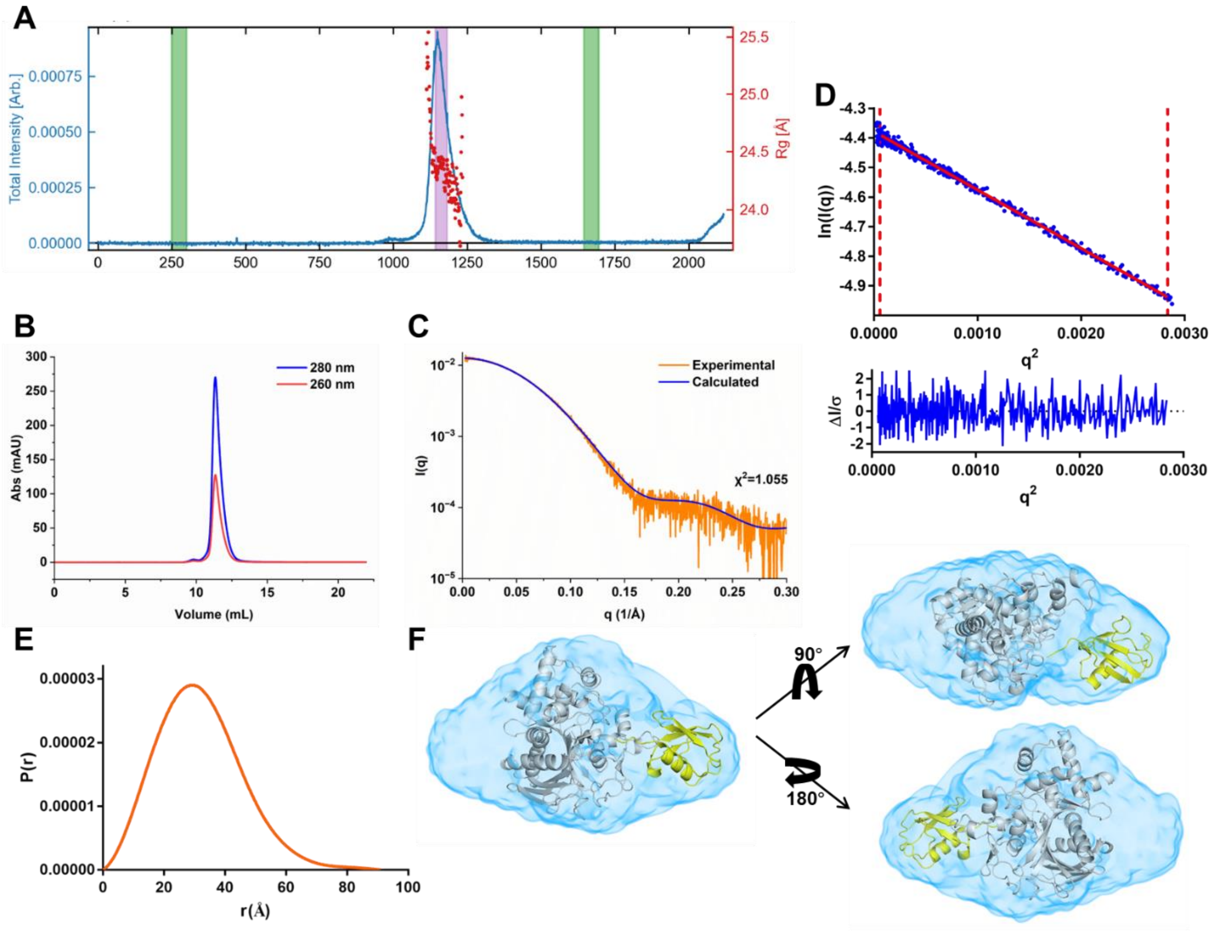

Supplementary Figure 5. SEC-SAXS analysis of MavL-UbVME in solution. (A) SAXS data summary showing series intensity and R_g vs. frame. Green shaded regions are buffer regions, purple shaded regions are sample regions. (B) Size exclusion profile of MavL-UbVME showing UV absorbance at 280 nm and 260 nm vs. elution volume. (C) Scattering curves of MavL-UbVME. Experimental curve is shown in orange, whereas back-calculated curve of MavL-UbVME (shown in F) crystal structure is shown in blue, with χ^2 marked on the graph. (D) Guinier analysis of low q values, ln(I(q)) (beam intensity) versus q2 (scattering angle). R_g of MavL-UbVME was shown in Table S3. (E) Pair distance distribution functions (P(r)) analysis with estimated maximum intramolecular distance (D_{max}). (F) 3D reconstruction of electron density suggests an elongated envelope of MavL-UbVME. Crystal structure of MavL-UbVME was used to fit the electron density. Unfitted density potentially accounts for C-terminal MavL residues not observed in crystal structures.

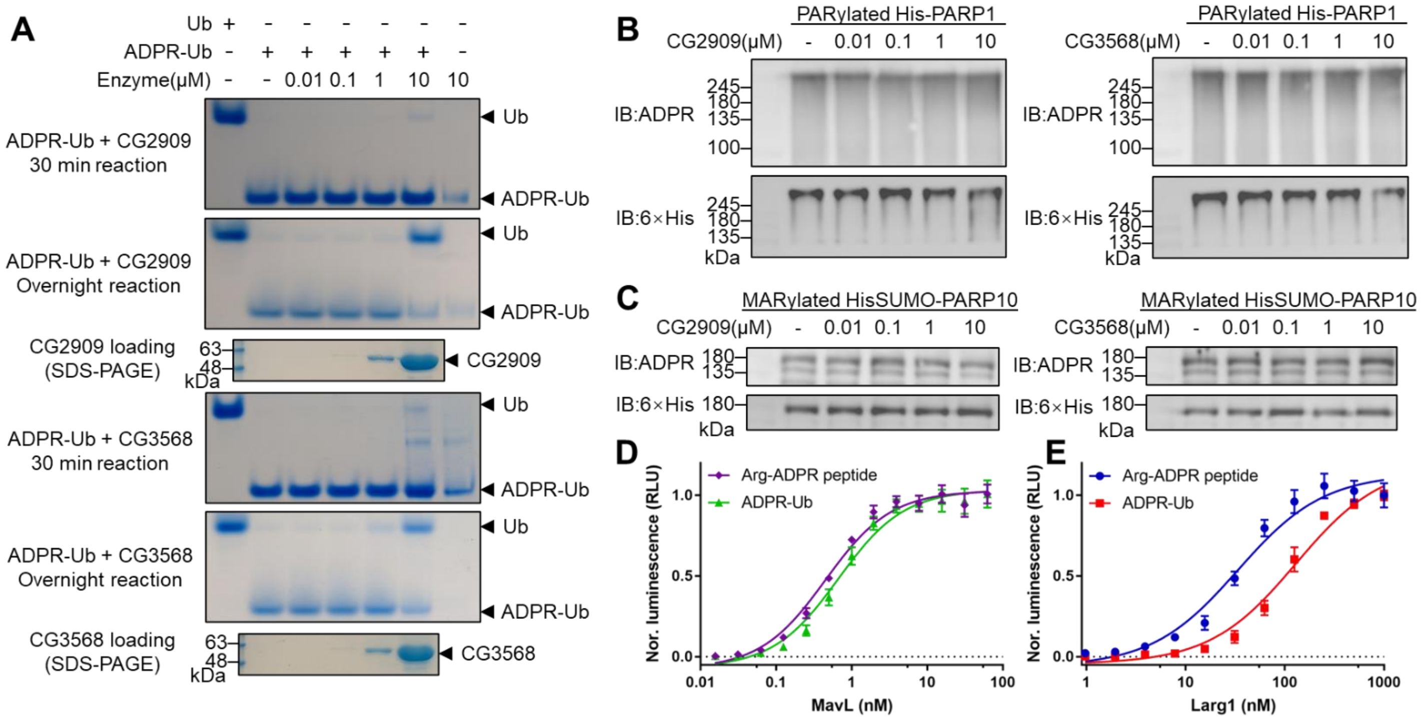

Supplementary Figure 6. Additional (ADP-ribosyl)hydrolysis assays. (A) Native PAGE showing (ADP-ribosyl)hydrolysis activities of CG2909 and CG3568 towards ADPR-Ub. Reactions were visualized by Coomassie Blue staining. Controls of Ub, ADPR-Ub, and enzymes alone were included. Loading of CG2909 and CG3568 were shown in SDS-PAGE gels. Experiments were performed three times independently with similar results. (B) In vitro de-PARylation assay of CG2909 and CG3568. Purified His-tagged PARP1 was auto-PARylated and incubated with varying concentrations of CG2909 and CG3568. Reactions were immunoblotted against ADPR to show the change in ADP-ribosylation level. Loading of the reactions was shown by immunoblotting against 6×His-tag. Experiments were performed three times independently with similar results. (C) In vitro de-MARylation assay of CG2909 and CG3568. Purified HisSUMO-tagged PARP10 was auto-MARylated and incubated with varying concentrations of CG2909 and CG3568. Reactions were immunoblotted against ADPR to show the change in ADP-ribosylation level. Loading of the reactions was shown by immunoblotting against 6×His-tag. Experiments were performed three times independently with similar results. (D) AMP-Glo assay measuring activity of MavL processing MARylated arginine peptide or ADPR-Ub. Error bars: standard deviation (SD) of the mean. (E) AMP-Glo assay measuring activity of Larg1 processing MARylated arginine peptide or ADPR-Ub. Error bars: standard deviation (SD) of the mean.

Supplementary Table 1. Crystallographic Data collection, processing, and refinement statistics.

	MavL$_{42\text{-}435}$	MavL$_{42\text{-}435}$-UbVME	MavL$_{42\text{-}435}$-UbVME (ADPR-bound form)	MavL$_{42\text{-}435}^{R370A}$ (ADPR-bound form)	CG2909$_{12\text{-}498}$ (ADPR-bound form)	CG3568$_{25\text{-}508}$ (ADPR-bound form)
**Wavelength (Å)**	0.9792	0.9792	0.9795	0.9792	0.9795	0.9793
**Resolution range (Å)**	39.31 - 2.17 (2.248 - 2.17)	69.71 - 2.195 (2.273 - 2.195)	39.1 - 2.15 (2.227 - 2.15)	33.78 - 1.86 (1.927 - 1.86)	38.49 - 2.28 (2.362 - 2.28)	92.25 - 2.0 (2.071 - 2.0)
**Space group**	P 1 21 1	P 31 2 1	P 31 2 1	P 41	P 1 21 1	P 21 21 21
**Unit cell**	93.232 157.244 117.621 90 96.201 90	108.719 108.719 209.143 90 90 120	108.957 108.957 209.546 90 90 120	95.557 95.557 54.046 90 90 90	48.949 90.006 212.267 90 95.892 90	104.048 129.805 199.433 90 90 90
**Total reflections**	841173 (85544)	757014 (72753)	157938 (15608)	188629 (18215)	154448 (11217)	1025701 (104719)
**Unique reflections**	176902 (17628)	73681 (7171)	78969 (7800)	41113 (4087)	77327 (5609)	182007 (17977)
**Multiplicity**	4.8 (4.9)	10.3 (10.1)	2.0 (2.0)	4.6 (4.5)	6.8 (6.6)	5.6 (5.8)
**Completeness (%)**	99.53 (99.65)	99.71 (98.41)	99.92 (99.73)	99.64 (99.71)	92.21 (67.03)	99.82 (99.93)
**Mean I/sigma(I)**	11.93 (1.72)	14.95 (2.35)	11.47 (2.17)	23.61 (2.49)	8.90 (1.64)	9.41 (1.87)
**Wilson B-factor**	39.69	39.79	33.17	34.84	28.77	28.33
**R-merge**	0.105 (1.082)	0.4104 (1.228)	0.04039 (0.3522)	0.04789 (0.615)	0.234 (1.922)	0.1097 (0.9599)
**CC1/2**	0.995 (0.559)	0.978 (0.725)	0.998 (0.765)	0.998 (0.781)	0.984 (0.658)	0.998 (0.744)
**Reflections (refinement)**	176690 (17627)	73647 (7171)	78945 (7798)	41045 (4087)	77255 (5610)	181778 (17968)
**Reflections (R-free)**	2000 (199)	3615 (384)	1995 (200)	2029 (193)	2026 (146)	8992 (841)
**R-work**	0.1875 (0.3079)	0.1777 (0.2366)	0.1730 (0.2593)	0.1574 (0.2333)	0.1953 (0.3121)	0.2321 (0.3066)
**R-free**	0.2145 (0.3385)	0.2095 (0.2706)	0.2069 (0.2630)	0.1843 (0.2651)	0.2359 (0.3784)	0.2730 (0.3542)
**RMS(bonds)**	0.003	0.009	0.004	0.011	0.003	0.008
**RMS(angles)**	0.67	1.02	0.74	1.14	0.60	0.94
**Ramachandran favored (%)**	95.32	95.68	97.05	95.15	95.88	95.96
**Ramachandran allowed (%)**	4.24	4.09	2.73	4.58	3.97	3.82
**Ramachandran outliers (%)**	0.44	0.23	0.23	0.27	0.15	0.21
**Average B-factor**	45.39	46.96	45.17	43.04	33.99	32.68

Supplementary Table 2. SEC-SAXS data collection and analysis

**SEC-SAXS Data collection**	
Instrument	BioCAT facility at the Advanced Photon Source beamline 18 ID
Detector	Eiger2 XE 9M
Wavelength (Å)	1.033
Camera length (m)	3.688
q-measurement range (1/Å)	0.0027–0.42
Exposure time (s)	0.5
Exposure period (s)	1.0
Column	Superdex 75 10/300 Increase
Buffer	1×PBS
Temperature (°C)	22
Sample loaded	300 µL at 5 mg/mL
**Data processing**	
Data reduction	BioXTAS RAW 2.1.4 (1) and ATSAS (2)
Guinier fit and molecular weight	BioXTAS RAW 2.1.4 (1)
P(r) function	GNOM (3)
3D reconstruction with electron density	DENSS (4)
Profile computation	CRYSOL (2)
**Guinier analysis and molecular weight**	
I(0)	0.0126±0.0000125
R_g (Å)	24.35±0.04
q range (1/Å)	0.00759–0.05326
Porod volume ($Å^3$)	58900
***P*(r) analysis**	
I(0)	0.0126±0.0000128
R_g (Å)	24.48±0.05
D_{max} (Å)	91
q range (1/Å)	0.007–0.3

Supplementary Table 3. Residue depth analysis on the distal ribose-interacting residues in DUF4804, PARG-like, and MacroD-type macrodomains. The PDB files of the structure were analyzed by the DEPTH web server to obtain information on residue depth. Residues interacting with the distal ribose were manually selected and analyzed. Glycine residues were excluded in calculating the average side chain depth. PDB code of the structures used in the analysis: (Larg1: 7W3S, *Tc*PARG: 3SIG, *Dr*PARG: 5ZDB, *Hs*PARG: 4B1H, Af1521: 2BFQ, MacroD1: 6LH4, MacroD2: 4IQY)

Class	Protein	Residue	Depth (Å)	Average depth (Å)	Backbone depth (Å)	Average backbone depth (Å)	Side chain depth (Å)	Average side chain depth (Å)
DUF4804	MavL	F227	7.84±1.34	8.36±1.08	6.64±0.66	8.43±0.78	8.40±0.87	8.18±0.74
		D315	10.62±1.10		11.61±0.38		9.54±0.56	
		N322	8.44±0.90		8.58±1.05		7.96±0.63	
		D323	6.00±0.91		6.52±0.70		5.24±0.76	
		D333	8.91±1.14		8.82±1.11		9.78±0.90	
	Larg1	F283	7.60±0.80	7.45±0.84	7.12±1.05	7.55±0.88	7.88±0.53	7.37±0.62
		D372	9.86±0.96		10.51±0.76		9.22±0.66	
		N379	6.88±0.73		7.05±0.99		6.72±0.43	
		E380	4.46±0.77		4.98±0.63		4.04±0.64	
		E387	8.59±0.95		8.10±0.96		8.99±0.82	
	CG2909	W342	5.82±1.06	7.12±0.98	4.99±0.32	7.43±0.66	6.15±1.07	6.81±0.64
		D432	9.33±1.29		10.24±0.99		8.43±0.83	
		N439	8.15±0.92		8.88±0.62		7.42±0.43	
		E440	6.13±0.99		7.09±0.42		5.36±0.39	
		T449	5.13±0.72		5.44±0.59		5.12±0.40	
		D451	8.16±0.87		7.94±1.03		8.38±0.75	
	CG3568	W355	6.30±1.24	7.22±0.99	4.98±0.05	7.42±0.63	6.83±1.07	6.95±0.65
		D441	9.78±1.32		10.74±0.95		8.82±0.85	
		N448	8.21±0.85		8.83±0.68		7.59±0.41	
		E449	5.98±0.97		6.88±0.48		5.26±0.51	
		T458	4.93±0.69		5.26±0.59		4.82±0.31	
		D460	8.13±0.89		7.86±1.05		8.39±0.74	
PARG-like	*Tc*PARG	E114	5.98±0.80	5.36±0.87	6.20±0.90	5.87±0.53	5.80±0.76	4.98±0.83
		E115	5.62±1.23		6.60±0.41		4.84±1.08	
		F227	4.49±0.58		4.80±0.29		4.31±0.64	
	*Dr*PARG	E111	3.26±0.22	3.83±0.41	3.33±0.25	4.06±0.32	3.20±0.20	3.67±0.38
		E112	3.52±0.26		3.66±0.29		3.40±0.20	
		F228	4.71±0.74		5.20±0.43		4.42±0.74	
	*Hs*PARG	E755	6.93±0.78	5.89±1.14	7.26±0.82	6.70±0.74	6.68±0.73	5.28±0.89
		E756	6.17±1.98		7.90±1.17		4.78±1.17	
		F875	4.58±0.67		4.95±0.24		4.37±0.76	
MacroD-type	Af1521	N34	5.08±0.97	5.42±0.75	5.78±0.67	5.46±0.45	4.38±0.67	6.05±0.65
		G41	3.72±0.36		3.72±0.36		N/A	
		V96	8.38±1.04		7.67±0.65		9.32±0.53	
		Y145	4.52±0.62		4.67±0.11		4.44±0.76	
	MacroD1	N174	4.30±0.83	4.60±0.52	4.97±0.60	4.82±0.32	3.63±0.22	4.59±0.49
		G182	3.83±0.24		3.83±0.24		N/A	
		D184	5.68±0.36		5.67±0.32		5.69±0.45	
		F272	4.59±0.65		4.82±0.10		4.45±0.80	
	MacroD1	N92	4.33±0.87	4.48±0.55	5.08±0.50	4.77±0.32	3.58±0.06	4.49±0.44
		G100	3.50±0.31		3.50±0.31		N/A	
		D102	5.66±0.37		5.72±0.29		5.59±0.48	
		F190	4.45±0.67		4.77±0.16		4.30±0.78	

Supplementary References

1. J. B. Hopkins, R. E. Gillilan, S. Skou, *BioXTAS RAW* : improvements to a free open-source program for small-angle X-ray scattering data reduction and analysis. *J Appl Crystallogr* **50**, 1545–1553 (2017).
2. D. Franke, *et al.*, *ATSAS 2.8* : a comprehensive data analysis suite for small-angle scattering from macromolecular solutions. *J Appl Crystallogr* **50**, 1212–1225 (2017).
3. D. I. Svergun, Determination of the regularization parameter in indirect-transform methods using perceptual criteria. *J Appl Crystallogr* **25**, 495–503 (1992).
4. T. D. Grant, Ab initio electron density determination directly from solution scattering data. *Nat Methods* **15**, 191–193 (2018).
