# Supplementary material for: Legionella metaeffector MavL reverses ubiquitin ADP-ribosylation via a conserved arginine-specific macrodomain
Source: Nat Commun. 2024 Mar 19;15:2452. doi: 10.1038/s41467-024-46649-2 (PMC10951314; doi:10.1038/s41467-024-46649-2)
Supplement: Supplementary file 3 — Supplementary Data 1 [file 41467_2024_46649_MOESM3_ESM.docx]

| Reagent or resource | source | identifier |
| --- | --- | --- |
| **antibody** |  |  |
| Mouse monoclonal anti-6×His | Proteintech | Cat# 66005 |
| Rabbit anti-pan-ADPR reagent | Sigma | Cat# MABE1016 |
| Mouse monoclonal anti-HA | Invitrogen | Cat# 26183 |
| Mouse monoclonal anti-Flag | Proteintech | Cat# 66008 |
| Rabbit monoclonal anti-β-actin | ABclonal | Cat# AC026 |
| Mouse monoclonal anti-β-tubulin | DSHB | Cat# E7 |
| Rabbit polyclonal anti-PGK | ABclonal | Cat# ab154613 |
| Rabbit polyclonal anti-UBE2Q1 | Invitrogen | Cat# PA5-70599 |
| Rabbit anti-SdeA | (1) |  |
| Rabbit anti-ICDH | (1) |  |
| Rabbit anti-GFP | (1) |  |
| Rabbit anti-*L. pneumophila* | (1) |  |
| **Bacterial strains** |  |  |
| *L. pneumophila Lp02* | (2) |  |
| *L. pneumophila Lp03* | (3) |  |
| *L. pneumophila Lp02 (ΔmavL)* | This paper |  |
| *L. pneumophila Lp02 (ΔmavL* pMavL*)* | This paper |  |
| *L. pneumophila Lp02 (ΔmavL* pMavL^D333A^*)* | This paper |  |
| *E. coli* DH5α | NEB | Cat# C2987 |
| *E. coli* BL21 (DE3) | Novagen | Cat# 70235 |
| *E. coli* Rosetta (DE3) | Novagen | Cat# 70954 |
| **Chemicals, Peptides, and Recombinant Proteins** |  |  |
| HA-Ub-Prg | Das lab |  |
| HA-Ub-VME | Das lab |  |
| HA-Ub-VS | South Bay Bio | Cat# SBB-PS0122 |
| Olaparib | Advanced ChemBlocks | Cat# F-4545 |
| Adenosine 5’-diphosphoribose sodium salt | Sigma | Cat# A0752 |
| β-Nicotinamide adenine dinucleotide | GoldBio | Cat# N-030 |
| EZview™ Red ANTI-FLAG® M2 Affinity Gel | Sigma | Cat# F2426 |
| Phospho-Tag™ Phosphoprotein Gel Stain | ABP Biosciences | Cat# P005A |
| MARylated arginine peptide | (4) |  |
| MARylated cysteine peptide | (5) |  |
| MARylated threonine peptide | (5) |  |
| MARylated serine peptide | (6) |  |
| MARylated mimetic histidine peptide | (7) |  |
| MARylated thymidine ssDNA | (8) |  |
| **Critical Commercial Assays** |  |  |
| AccuPower® ProFi Taq PCR PreMix | Bioneer | Cat# K-2632 |
| Pierce™ BCA Protein Assay Kit | Thermo Scientific | Cat# 23227 |
| AMP-Glo™ Assay | Promega | Cat# V5011 |
| **Deposited data** |  |  |
| Structure of apo MavL | (9) | PDB: 6OMI |
| Structure of ADPR-bound TARG1 | (10) | PDB: 4J5S |
| Structure of ADPR-bound DarG | (11) | PDB: 5M3E |
| Structure of apo *Tc*PARG | (12) | PDB: 3SIH |
| Structure of ADPR-bound Larg1 | (13) | PDB: 7W3S |
| Structure of apo MavL_42-435_ | This paper | PDB: 8DMP |
| Structure of MavL_42-435_-UbVME | This paper | PDB: 8DMQ |
| Structure of ADPR-bound MavL_42-435_-UbVME | This paper | PDB: 8DMS |
| Structure of ADPR-bound MavL_42-435_^R370A^ | This paper | PDB: 8DMR |
| Structure of ADPR-bound CG2909_12-498_ | This paper | PDB: 8DMT |
| Structure of ADPR-bound CG3568^25-508^ | This paper | PDB: 8DMU |
| **Recombinant DNA** |  |  |
| Plasmid: pGEX-6P-1-MavL_42-435_ | This paper |  |
| Plasmid: pGEX-6P-1-MavL_42-435_ E107A | This paper |  |
| Plasmid: pGEX-6P-1-MavL_42-435_ H142A/Y265A | This paper |  |
| Plasmid: pGEX-6P-1-MavL_42-435_ F227A | This paper |  |
| Plasmid: pGEX-6P-1-MavL_42-435_ K236A | This paper |  |
| Plasmid: pGEX-6P-1-MavL_42-435_ D315A | This paper |  |
| Plasmid: pGEX-6P-1-MavL_42-435_ N322A | This paper |  |
| Plasmid: pGEX-6P-1-MavL_42-435_ D323A | This paper |  |
| Plasmid: pGEX-6P-1-MavL_42-435_ T331A | This paper |  |
| Plasmid: pGEX-6P-1-MavL_42-435_ D332A | This paper |  |
| Plasmid: pGEX-6P-1-MavL_42-435_ D333A | This paper |  |
| Plasmid: pGEX-6P-1-MavL_42-435_ R370A | This paper |  |
| Plasmid: pGEX-6P-1-MavL_42-435_ E75L | This paper |  |
| Plasmid: pGEX-6P-1-MavL_42-435_ E75R | This paper |  |
| Plasmid: pGEX-6P-1-MavL_42-435_ F105A | This paper |  |
| Plasmid: pGEX-6P-1-MavL_42-435_ F105D | This paper |  |
| Plasmid: pGEX-6P-1-MavL_42-435_ Q109L | This paper |  |
| Plasmid: pGEX-6P-1-MavL_42-435_ Q109R | This paper |  |
| Plasmid: pGEX-6P-1-MavL_42-435_ Y111L | This paper |  |
| Plasmid: pGEX-6P-1-MavL_42-435_ D113R | This paper |  |
| Plasmid: pGEX-6P-1-MavL_42-435_ T115V | This paper |  |
| Plasmid: pGEX-6P-1-MavL_42-435_ F105A/Q109A/Y111A/D113A | This paper |  |
| Plasmid: pGEX-6P-1-MavL_42-435_ Q109A/Y111A/D113A | This paper |  |
| Plasmid: pRSET-A-Ub | Das lab |  |
| Plasmid: pRSET-A-Ub^Q40E^ | Das lab |  |
| Plasmid: pET-DUET1-Ub^Q40L^ | Das lab |  |
| Plasmid: pET-DUET1-Ub^E51K^ | Das lab |  |
| Plasmid: pET-DUET1-Ub^E51L^ | Das lab |  |
| Plasmid: pET-DUET1-Ub^D52L^ | Das lab |  |
| Plasmid: pET28a-Ub | Das lab |  |
| Plasmid: pTXB1-Ub_1-75_-intein-CBD | Das lab |  |
| Plasmid: pTXB1-HA-Ub | This paper |  |
| Plasmid: pGEX-6P-1-DupA | This paper |  |
| Plasmid: pGEX-6P-1-DupB | This paper |  |
| Plasmid: pET41a-*h*ARH1 | (14) |  |
| Plasmid: pGEX-6P-1-Larg1 | This paper |  |
| Plasmid: pGEX-6P-1-SdeA_519-1100_ (mART) | (15) |  |
| Plasmid: pGEX-6P-1-SdeA_178-1000_ (mART+PDE) | (15) |  |
| Plasmid: pGEX-6P-1-CteC | (16) |  |
| Plasmid: pET28b-PARP1 | Gifted by Prof. Michael Cohen |  |
| Plasmid: pET-His-SUMO-PARP10 | Gifted by Prof. Michael Cohen |  |
| Plasmid: pGEX-4T-1-*Hs*PARG_446-966_ | Gifted by Prof. Michael Cohen |  |
| Plasmid: pGEX-4T-1-MacroD2 | Gifted by Prof. Michael Cohen |  |
| Plasmid: pGEX-6P-1-Gdp2h | This paper |  |
| Plasmid: pGEX-6P-1-Lart1 | This paper |  |
| Plasmid: pET15b-Rab5(ΔCAAX) | (17) |  |
| Plasmid: pET15b-ExoS_78-453_ | (17) |  |
| Plasmid: pET15b-14-3-3 | (17) |  |
| Plasmid: pGEX-6P-1-CG2909 | Gifted by Prof. Sokol Todi |  |
| Plasmid: pGEX-6P-1-CG3568 | Gifted by Prof. Sokol Todi |  |
| Plasmid: pGEX-6P-1-CG2909_12-498_ | This paper |  |
| Plasmid: pGEX-6P-1-CG3568_25-508_ | This paper |  |
| Plasmid: pEGFP-C1-MavL | This paper |  |
| Plasmid: pEGFP-C1-MavL D333A | This paper |  |
| Plasmid: pAPH-HA-MavL | This paper |  |
| Plasmid: pAPH-HA-MavL_41-455_ | This paper |  |
| Plasmid: pFlag-CMV2-SdeA | (15) |  |
| Plasmid: pFlag-CMV2-Rab33b | (15) |  |
| Plasmid: pAPH-HA-Ub^AA^ | (15) |  |
| Plasmid: pFlag-CMV2-UBE2Q1 | This paper |  |
| Plasmid: pFlag-CMV2-UBE2Q1_1-214_ | This paper |  |
| Plasmid: pFlag-CMV2-UBE2Q1_215-422_ | This paper |  |
| Plasmid: pFlag-CMV2-UBE2Q1_38-177_ | This paper |  |
| Plasmid: pGEX-6P-1-UBE2Q1 | This paper |  |
| Plasmid: pYES1NTA-SdeA | (15) |  |
| Plasmid: pYES1NTA-SdeA H227A | (15) |  |
| Plasmid: p425GPD-MavL | This paper |  |
| Plasmid: p425GPD-MavL D333A | This paper |  |
| Plasmid: pSR47S-*ΔmavL* | This paper |  |
| **Software and algorithms** |  |  |
| MaxQuant | (18) | https://www.maxquant.net/ |
| Pro-Origami | (19) | http://munk.cis.unimelb.edu.au/pro-origami/ |
| TopDraw | (20) | https://bondxray.org/software/topdraw/topdraw.html |
| PyMOL | Schrödinger, LLC | http://www.pymol.org |
| COOT | (21) | https://www2.mrc-lmb.cam.ac.uk/personal/pemsley/coot/ |
| CCP4i2 | (22) | https://www.ccp4.ac.uk/ |
| PHENIX | (23) | http://www.phenix-online.org/ |
| HKL3000 | (24) | https://www.hkl-xray.com/hkl-3000 |
| XDS | (25) | https://xds.mr.mpg.de/ |
| HMMER | (26) | https://www.ebi.ac.uk/Tools/hmmer/ |
| RosettaLigand | (27) | https://rosie.graylab.jhu.edu/ligand_docking |
| DEPTH | (28) | http://cospi.iiserpune.ac.in/depth |
| ChemDraw 20.0 | Perkin Elmer Informatics | https://perkinelmerinformatics.com/products/research/chemdraw |
| GraphPad Prism 7.0 | GraphPad Software | https://www.graphpad.com/ |

**References**
