# Supplementary material for: Legionella metaeffector MavL reverses ubiquitin ADP-ribosylation via a conserved arginine-specific macrodomain
Source: Nat Commun. 2024 Mar 19;15:2452. doi: 10.1038/s41467-024-46649-2 (PMC10951314; doi:10.1038/s41467-024-46649-2)
Supplement: Supplementary file 4 — Supplementary Data 2 [file 41467_2024_46649_MOESM4_ESM.docx]

| Primer | Sequence (5’ to 3’) |
| --- | --- |
| MavL_F_BamHI | ctgggatccATGAGATTGTTTCAGTACAA |
| MavL_R_XhoI | cgtctcgagTTACTGAGGACCCGATTTTT |
| MavL_42_F_BamHI | catggatccgcctatcaattgttgctcagtaaagaaacc |
| MavL_435_R_XhoI | catctcgagTTAactatgagaaaaaagactgaacatactacgc |
| MavL-D332A-For | cgtcaaacagctgatggtgtcaaggcc |
| MavL-D332A-Rev | ggccttgacaccatcagctgtttgacg |
| MavL-D333A-For | cgtcaaacagatgctggtgtcaaggcc |
| MavL-D333A-Rev | ggccttgacaccagcatctgtttgacg |
| MavL-F227A-For | ggtataggaacagggtgtgcttcaggcgcttattatg |
| MavL-F227A-Rev | cataataagcgcctgaagcacaccctgttcctatacc |
| MavL-E212A-For | cgcgacaaaatgcaaagcaggcagc |
| MavL-E212A-Rev | gctgcctgctttgcattttgtcgcg |
| MavL-H277A-For | gctgaaaagaagattggtgctatgtcttttcgtgtgagtc |
| MavL-H277A-Rev | gactcacacgaaaagacatagcaccaatcttcttttcagc |
| MavL-C226A-For | ggtataggaacagggGCtttttcaggcgcttattatg |
| MavL-C226A-Rev | cataataagcgcctgaaaaaGCccctgttcctatacc |
| MavL-E107A-F | cacaggtttttgctgCaagtcaggtttatcatg |
| MavL-E107A-R | catgataaacctgacttGcagcaaaaacctgtg |
| MavL-H142A-F | gtacaatgatggtggaGCtggctcctctttcaag |
| MavL-H142A-R | cttgaaagaggagccaGCtccaccatcattgtac |
| MavL-Y265A-F | cattcattatgaccctGCtatgggagatgaacctg |
| MavL-Y265A-R | caggttcatctcccataGCagggtcataatgaatg |
| MavL-D315A-F | caattgttgcctgggCtcatttttcctg |
| MavL-D315A-R | caggaaaaatgaGcccaggcaacaattg |
| MavL-N322A-F | cctggcccggaGCtgattattgggg |
| MavL-N322A-R | ccccaataatcaGCtccgggccagg |
| MavL-D323A-F | cctggcccggaaatgCttattgggg |
| MavL-D323A-R | ccccaataaGcatttccgggccagg |
| MavL-F105A-F | gtctaaaaaaccacaggttGCtgctgaaagtcagg |
| MavL-F105A-R | cctgactttcagcaGCaacctgtggttttttagac |
| MavL-T331A-F | ggtgctcgtcaaGcagatgatgg |
| MavL-T331A-R | ccatcatctgCttgacgagcacc |
| MavL_R370A_F | ccagaatcatttactaaagacGCtaaaggtatgagtgactggg |
| MavL_R370A_R | cccagtcactcatacctttaGCgtctttagtaaatgattctgg |
| MavL_K236A_F | cgcttattatgatgtgattGCaccctatgtcagaaacgc |
| MavL_K236A_R | gcgtttctgacatagggtGCaatcacatcataataagcg |
| MavL_T224A_F | cccggtataggaGcagggtgtttttc |
| MavL_T224A_R | gaaaaacaccctgCtcctataccggg |
| MavL_FQDY_4A_F | CCACAGGTTGCTGCTGAAAGTGCGGTTGCTCATGCTGGAACTGACTGGACACTGG |
| MavL_FQDY_4A_R | CCAGTGTCCAGTCAGTTCCAGCATGAGCAACCGCACTTTCAGCAGCAACCTGTGG |
| MavL_QDY_3A_F | GGTTTTTGCTGAAAGTGCGGTTGCTCATGCTGGAACTGACTGGACAC |
| MavL_QDY_3A_R | GTGTCCAGTCAGTTCCAGCATGAGCAACCGCACTTTCAGCAAAAACC |
| MavL_F105D_F | CTAAAAAACCACAGGTTGATGCTGAAAGTCAGG |
| MavL_F105D_R | CCTGACTTTCAGCATCAACCTGTGGTTTTTTAG |
| MavL_Q109L_F | GGTTTTTGCTGAAAGTCTGGTTTATCATGATG |
| MavL_Q109L_R | CATCATGATAAACCAGACTTTCAGCAAAAACC |
| MavL_Q109R_F | GGTTTTTGCTGAAAGTCGGGTTTATCATGATG |
| MavL_Q109R_R | CATCATGATAAACCCGACTTTCAGCAAAAACC |
| MavL_Y111L_F | GCTGAAAGTCAGGTTCTTCATGATGGAACTG |
| MavL_Y111L_R | CAGTTCCATCATGAAGAACCTGACTTTCAGC |
| MavL_D113R_F | GTCAGGTTTATCATCGTGGAACTGACTGGACAC |
| MavL_D113R_R | GTGTCCAGTCAGTTCCACGATGATAAACCTGAC |
| MavL_T115V_F | GGTTTATCATGATGGAGTTGACTGGACACTGG |
| MavL_T115V_R | CCAGTGTCCAGTCAACTCCATCATGATAAACC |
| MavL_E75R_F | CACGCCCGGTAAATTTTTTCTTCGAGAATTAAGTAAACAAG |
| MavL_E75R_R | CTTGTTTACTTAATTCTCGAAGAAAAAATTTACCGGGCGTG |
| MavL_E75L_F | CACGCCCGGTAAATTTTTTCTTCTAGAATTAAGTAAACAAG |
| MavL_E75L_R | CTTGTTTACTTAATTCTAGAAGAAAAAATTTACCGGGCGTG |
| CG2909_12_F_BamHI | CATGGATCCTGGCCAGCGGTCACC |
| CG2909_498_R_XhoI | CATCTCGAGTCAAATCACCCGCTTGGCATATTC |
| CG3568_25 _F | CCAGGGGCCCCTGGGATCCTGGCCTGGTAAACGTACAAATC |
| CG3568_R | GCGGCCGCTCGAGTCACACAATCAGTCGCCTAGC |
| UBE2Q1_F_HindIII | CATAAGCTTATGCAGCAGCCGC |
| UBE2Q1_F_EcoRI | CATGAATTCATGCAGCAGCCGC |
| UBE2Q1_R_NotI | CATGCGGCCGCCTAGCCGTCTTCTTTTGGGG |
| UBE2Q1_214_R_NotI | CATGCGGCCGCCTAGCCCTCAGCTGGCTC |
| UBE2Q1_215_F_HindIII | CATAAGCTTAAGAAATCTGAAGATGATGGCATTGG |
| UBE2Q1_38_F_BglII | CATAGATCTGGGCCCTGCCTGAGG |
| UBE2Q1_38_F_HindIII | CATAAGCTTGGGCCCTGCCTGAGG |
| UBE2Q1_177_R_EcoRI | CATGAATTCCTATGCTGGCAAGGGTTGATC |
| UBE2Q1_F_BglII | CATAGATCTATGCAGCAGCCGCAG |
| UBE2Q1_R_salI | CATGTCGACCTAGCCGTCTTCTTTTGGGG |
| Larg1_BamHI_F | CATGGATCCatgcggtctaaatttttttcatttttcac |
| Larg1_XhoI_R | CATCTCGAGctagagtttaatgctcgatgaggattg |
| YeastGDH2_SmaI_F | CATCCCGGGTATGCTTTTTGATAACAAAAATCGCGG |
| YeastGDH2_NotI_R | CATGCGGCCGCTCAAGCACTTGCCTCCGC |
| lpg0181_SmaI_F | CATCCCGGGTatgtatagtaaatatccagcatttttcctaaataag |
| lpg0181_NotI_R | CATCTCGAGctaatcataaactggctttgcccag |
| mavl_up_1.2kb_SalI_F | catgtcgacGAAGGTTATCACAGCTACAGTGAGG |
| mavl_up_1.2kb_BamHI_R | catggatccTAAAATTTTATTCAGGGTTTCTTTACTGAGCAAC |
| mavl_down_1.2kb_BamHI_F | catggatccTCTCCAACTGTTAAAGAGGAAGAATCG |
| mavl_down_1.2kb_NotI_R | catgcggccgcTTGGTTTTTTATCAGAATGCGCTG |
| Ub_D39L_F | GGCATCCCTCCTCTCCAGCAGAGG |
| Ub_D39L_R | CCTCTGCTGGAGAGGAGGGATGCC |
| Ub_Q40E_F | CCCTCCTGACGAGCAGAGGTTG |
| Ub_Q40E_R | CAACCTCTGCTCGTCAGGAGGG |
| Ub_Q40L_F | CCTCCTGACCTGCAGAGGTTG |
| Ub_Q40L_R | CAACCTCTGCAGGTCAGGAGG |
| Ub_E51K_F | GGAAACAGCTGAAAGATGGACGCAC |
| Ub_E51K_R | GTGCGTCCATCTTTCAGCTGTTTCC |
| Ub_E51L_F | GGAAACAGCTGTTAGATGGACGCACC |
| Ub_E51L_R | GGTGCGTCCATCTAACAGCTGTTTCC |
| Ub_D52L_F | GAAACAGCTGGAACTTGGACGCACCC |
| Ub_D52L_R | GGGTGCGTCCAAGTTCCAGCTGTTTC |
