# Supplementary material for: Legionella metaeffector MavL reverses ubiquitin ADP-ribosylation via a conserved arginine-specific macrodomain
Source: Nat Commun. 2024 Mar 19;15:2452. doi: 10.1038/s41467-024-46649-2 (PMC10951314; doi:10.1038/s41467-024-46649-2)
Supplement: Supplementary file 6 — Reporting Summary [file 41467_2024_46649_MOESM6_ESM.pdf]

nature portfolio

Corresponding author(s): Chittaranjan Das

Last updated by author(s): Jan 31, 2024

Reporting Summary

Nature Portfolio wishes to improve the reproducibility of the work that we publish. This form provides structure for consistency and transparency in reporting. For further information on Nature Portfolio policies, see our Editorial Policies and the Editorial Policy Checklist.

Statistics

For all statistical analyses, confirm that the following items are present in the figure legend, table legend, main text, or Methods section.

n/a	Confirmed	
☐	☒	The exact sample size (n) for each experimental group/condition, given as a discrete number and unit of measurement
☐	☒	A statement on whether measurements were taken from distinct samples or whether the same sample was measured repeatedly
☐	☒	The statistical test(s) used AND whether they are one- or two-sided<br>*Only common tests should be described solely by name; describe more complex techniques in the Methods section.*
☒	☐	A description of all covariates tested
☐	☒	A description of any assumptions or corrections, such as tests of normality and adjustment for multiple comparisons
☐	☒	A full description of the statistical parameters including central tendency (e.g. means) or other basic estimates (e.g. regression coefficient) AND variation (e.g. standard deviation) or associated estimates of uncertainty (e.g. confidence intervals)
☐	☒	For null hypothesis testing, the test statistic (e.g. F, t, r) with confidence intervals, effect sizes, degrees of freedom and P value noted<br>*Give P values as exact values whenever suitable.*
☒	☐	For Bayesian analysis, information on the choice of priors and Markov chain Monte Carlo settings
☒	☐	For hierarchical and complex designs, identification of the appropriate level for tests and full reporting of outcomes
☒	☐	Estimates of effect sizes (e.g. Cohen's d, Pearson's r), indicating how they were calculated

Our web collection on statistics for biologists contains articles on many of the points above.

Software and code

Policy information about availability of computer code

Data collection	No software was used.
Data analysis	Proteomics datasets were analyzed by MaxQuant 1.6.17.0. ITC data were integrated and analyzed by MicroCal PEAQ-ITC Analysis Software v1.41 (Malvern Panalytical). X-ray diffraction data were scaled and integrated by HKL3000 or XDS. Phenix, COOT, and CCP4i were used for protein structure building and refinements. The docking of Arg-ADPR and MavL was performed using RosettaLigand web server. The SEC-SAXS dataset was processed by BioXTAS RAW 2.1.4, ATSAS, GNOM, CRYSOL, and DENSS. The topology diagrams were generated using TopDraw and Pro-origami. Homology search was performed using HMMER server and the HMM logos were generated by Skylign web server . The residue depth analysis was performed using the DEPTH web server.

For manuscripts utilizing custom algorithms or software that are central to the research but not yet described in published literature, software must be made available to editors and reviewers. We strongly encourage code deposition in a community repository (e.g. GitHub). See the Nature Portfolio guidelines for submitting code & software for further information.

Data

Policy information about availability of data

All manuscripts must include a data availability statement. This statement should provide the following information, where applicable:

- Accession codes, unique identifiers, or web links for publicly available datasets
- A description of any restrictions on data availability
- For clinical datasets or third party data, please ensure that the statement adheres to our policy

Structural factors and atomic coordinates of MavL42-435, MavL42-435-UbVME, ADPR-bound MavL42-435-UbVME, ADPR-bound MavL42-435R370A, ADPR-bound CG290912-498, and ADPR-bound CG356825-508 have been deposited to Protein Data Bank with accession codes 8DMP [https://doi.org/10.2210/pdb8dmp/pdb], 8DMQ [https://doi.org/10.2210/pdb8dmq/pdb], 8DMS [https://doi.org/10.2210/pdb8dms/pdb], 8DMR [https://doi.org/10.2210/pdb8dmr/pdb], 8DMT [https://doi.org/10.2210/pdb8dmt/pdb], and 8DMU [https://doi.org/10.2210/pdb8dmu/pdb]. Proteomics dataset identifying Ub-interacting Legionella effectors has been deposited to Mass Spectrometry Interactive Virtual Environment (MassIVE) with the accession code MSV000093623 [https://massive.ucsd.edu/ProteoSAFe/dataset.jsp?accession=MSV000093623]. Legionella pneumophila reference proteome was obtained from Uniprot (Uniprot proteome ID: UP000000609). Other data, including full gels, blots, and raw data used to generate plots, are provided in the Source Data file.

Research involving human participants, their data, or biological material

Policy information about studies with human participants or human data. See also policy information about sex, gender (identity/presentation), and sexual orientation and race, ethnicity and racism.

Reporting on sex and gender	N/A
Reporting on race, ethnicity, or other socially relevant groupings	N/A
Population characteristics	N/A
Recruitment	N/A
Ethics oversight	N/A

Note that full information on the approval of the study protocol must also be provided in the manuscript.

Field-specific reporting

Please select the one below that is the best fit for your research. If you are not sure, read the appropriate sections before making your selection.

- [x] Life sciences
- [ ] Behavioural & social sciences
- [ ] Ecological, evolutionary & environmental sciences

For a reference copy of the document with all sections, see nature.com/documents/nr-reporting-summary-flat.pdf

Life sciences study design

All studies must disclose on these points even when the disclosure is negative.

Sample size	No sample size calculation was performed. The sample size chosen in this study was commonly used in similar experiments, as validated by previously published literatures.
Data exclusions	No data were excluded from analyses.
Replication	All experiments were performed in biological triplicates, with success, to validate the reproducibility of the findings.
Randomization	Not relevant to this study, as no grouping is applied in this study.
Blinding	Not relevant to this study, as no grouping is applied in this study.

Reporting for specific materials, systems and methods

We require information from authors about some types of materials, experimental systems and methods used in many studies. Here, indicate whether each material, system or method listed is relevant to your study. If you are not sure if a list item applies to your research, read the appropriate section before selecting a response.

Materials & experimental systems

n/a	Involved in the study
☐	☒ Antibodies
☐	☒ Eukaryotic cell lines
☒	☐ Palaeontology and archaeology
☒	☐ Animals and other organisms
☒	☐ Clinical data
☒	☐ Dual use research of concern
☒	☐ Plants

Methods

n/a	Involved in the study
☒	☐ ChIP-seq
☒	☐ Flow cytometry
☒	☐ MRI-based neuroimaging

Antibodies

Antibodies used

Anti-6×His: Proteintech Cat# 66005; Anti-pan-ADPR reagent: Sigma Cat# MABE1016; Anti-HA: Invitrogen Cat# 26183; Anti-Flag: Proteintech Cat# 66008; Anti-β-actin: ABclonal Cat# AC026; Anti-β-tubulin: DSHB Cat# E7; Anti-PGK: ABclonal Cat# ab154613; Anti-UBE2Q1: Invitrogen Cat# PA5-70599; Anti-SdeA, anti-ICDH, anti-GFP, and anti-L. pneumophila antibodies were produced by Pocono Rabbit Farm and Laboratory, Canadensis, PA.

Validation

Anti-6×His: His-tagged fusion protein, validated by vendor.
Anti-pan-ADPR reagent: ADPR-PARP1, validated by vendor.
Anti-HA: HA-H3 and p65-HA, validated by vendor.
Anti-Flag: Flag-TDP-43, validated by vendor.
Anti-β-actin: β-actin, validated by vendor.
Anti-β-tubulin: β-tubulin, validated by vendor.
Anti-PGK: PGK1, validated by vendor.
Anti-UBE2Q1: UBE2Q1, validated by vendor.
Anti-SdeA antibody was described in Qiu et al. Nature. 2016;533:120-124.
Anti-ICDH, anti-GFP, and anti-L. pneumophila antibodies were described in Xu et al. PLoS Pathog. 2010;6(3):e1000822.

Eukaryotic cell lines

Policy information about cell lines and Sex and Gender in Research

Cell line source(s)

HEK293T (ATCC, CRL-3216), RAW 264.7 (ATCC, TIB-71)

Authentication

Not authenticated. All cell lines were purchased from ATCC.

Mycoplasma contamination

All the cell lines were tested negative for mycoplasma contamination.

Commonly misidentified lines
(See ICLAC register)

No commonly misidentified line was used.

Plants

Seed stocks

N/A

Novel plant genotypes

N/A

Authentication

N/A
